# On Compression of the Aorta as a Remedy for Uterine Hemorrhage after Delivery

**Published:** 1844-06

**Authors:** Daniel Brainard

**Affiliations:** Chicago


*On Compression of the Aorta as a remedy for Uterine Hemorrhage after delivery.* By Daniel Brainard, M. D.

*Dec.* 11, 1843, *at* 1½ *o'clock P. M.*, I was called to visit Mrs. E. D. G., a highly respectable lady of this city, in labor with her first child. She is a person of delicate habit, and was subject, during several years, to a "spinal complaint" and loss of motion of her limbs, which of late has disappeared, and she has enjoyed good health. On examination, found the pelvis well formed, the vertex presenting, os uteri thick and rigid and little dilated, pains severe and regular. At 7 o'clock P. M., the os uteri began to dilate; and at 1 o'clock on the morning of the 12th, a full sized male child was delivered, the placenta immediately following. At this time I placed my hand upon the abdomen, and felt the uterus contracted.

In about fifteen minutes the patient complained of faintness; and on going to the bedside, I perceived that a profuse hemorrhage had occurred. The use of cold applications and pressure upon the abdomen were immediately resorted to, but the uterus was flaccid, and only contracted from time to time sufficiently to throw off the coagula. The *tampon* was carefully introduced, and Tinct. Ergot, 2 drachms, administered every twenty minutes until 6 drachms had been given. The flowing being still profuse, the fainting constant, with pallor and coldness of the skin, I became alarmed for her safety, and determined to apply pressure upon the abdominal aorta, and was somewhat surprised to find that it could be effected so as entirely to interrupt the circulation in the vessel, without the slightest inconvenience, or pain to the patient. The intestines having been pressed upward and backward by the gravid uterus, when the latter contracted, a space was left in which the vessel might be compressed with much greater facility than can be the femoral artery, where it passes over the pubis.

The immediate effect of the application was as delightful as possible; in about twenty seconds the pulsations, from having been weak and scarcely perceptible, became regular and strong, the flowing ceased, color returned to the face, she said she felt well, and seemed quite unconscious of having been in a state of prolonged syncope. The nausea, before present, subsided and she asked for food. The compression was made with the ends of the fingers, the knuckles, a wallet, or the ulnar side of the hand; was continued one and a half hours without interruption, excepting for a few seconds, on changing the mode of making it, when the patient relapsed into a state of syncope, but immediately revived on its re-application: at the end of this time the uterus was contracted so that it was discontinued.

Pressure of the aorta as a remedy for uterine hemorrage after delivery, or in cases of syncope, was, it is said, first suggested by the elder Baudelocque, but it is only of late that it has been reckoned as one of the resources of art against these accidents, by the profession at large. My object in offering the above case to the notice of the profession, is not only to make it known to them, but also to recommend its use *in the first instance*, and in connexion with frictions, pressure upon the uterine region, &c., instead of deferring it, as was done in this case, until all other remedies had been tried in vain. In cases of protracted syncope, the system is insensible to the action of astringents, ergot, &c., the stomach is often nauseated; in these cases compression restores the functions of the nervous system, and relieves the nausea, thereby enabling us to obtain the action of other remedies for the permanent relief of the accident. The above case will illustrate the good effects of this means, in this state of the system, especially when resulting from loss of blood; the manner in which it acts, and the cases in which it is applicable, will, however, readily suggest themselves to the reflecting practitioner, and need not therefore be further dwelt upon in the present paper.

Chicago, June 1, 1844.

